# New drugs in psychiatry: focus on new pharmacological targets

**DOI:** 10.12688/f1000research.10233.1

**Published:** 2017-03-30

**Authors:** Filippo Caraci, Gian Marco Leggio, Salvatore Salomone, Filippo Drago

**Affiliations:** 1 Department of Drug Sciences, University of Catania, Catania, Italy; 2 IRCCS Associazione Oasi Maria S.S., Institute for Research on Mental Retardation and Brain Aging, Troina (EN), Italy; 3 Department of Biomedical and Biotechnological Sciences, University of Catania, Catania, Italy

**Keywords:** psychotropic drugs, cariprazine, vortioxetine, ketamine oresketamine, aducanumab, neuropsychopharmacology, anti-psychotic

## Abstract

The approval of psychotropic drugs with novel mechanisms of action has been rare in recent years. To address this issue, further analysis of the pathophysiology of neuropsychiatric disorders is essential for identifying new pharmacological targets for psychotropic medications. In this report, we detail drug candidates being examined as treatments for psychiatric disorders. Particular emphasis is placed on agents with novel mechanisms of action that are being tested as therapies for depression, schizophrenia, or Alzheimer’s disease. All of the compounds considered were recently approved for human use or are in advanced clinical trials. Drugs included here are new antipsychotic medications endowed with a preferential affinity at dopamine D3 receptor (cariprazine) or at glutamatergic or cannabinoid receptors, as well as vortioxetine, a drug approved for managing the cognitive deficits associated with major depression. New mechanistic approaches for the treatment of depression include intravenous ketamine or esketamine or intranasal esketamine. As for Alzheimer’s disease, the possible value of passive immunotherapy with agents such as aducanumab is considered to be a potential disease-modifying approach that could slow or halt the progressive decline associated with this devastating disorder.

## Introduction

Epidemiological data indicate neuropsychiatric disorders as being some of the most prevalent, devastating, and yet poorly treated illnesses. As the approval of central nervous system drugs with novel mechanisms of action has been rare in recent years, there is a critical need to enhance drug discovery in neuropsychopharmacology ^1^. To achieve this goal, it is essential to focus on developing drugs that target the pathophysiology underlying the disease, which increases the likelihood of identifying efficacious agents rather than symptomatic treatments. The pathology-to-drug discovery approach specifies that an understanding of the pathophysiology of neuropsychiatric disorders is the required initial step in identifying disease pathways and for validating new pharmacological targets ^1^. A more complete understanding of the disease pathways will facilitate both the selection of therapeutic targets and the development of relevant models for screening drug candidates ^2^. The pathology-to-drug discovery approach inspired the creation of NEWMEDS (Novel Methods Leading to New Medications in Depression and Schizophrenia), a European project designed to identify specific brain circuits, particularly those involving the prefrontal cortex, that are involved in the pathophysiology and treatment of major depression and schizophrenia ^3^. On the other hand, a better knowledge of the mechanism of action of older drugs (the so-called reverse translation approach) has permitted, in part, a better understanding of the pathophysiology of neuropsychiatric diseases, enabling drug design according to the pathology-to-drug discovery approach.

As the neuropsychopharmacology community has only recently adopted the pathology-to-drug discovery approach, it remains unknown whether agents discovered in this way are currently available for the clinical management of psychiatric disorders and whether such drugs display significant advantages over conventional therapies with respect to efficacy and tolerability.

Summarized below are some of the more exciting and relevant advances in the field of neuropsychopharmacology as they pertain to the design and development of novel psychotherapeutics. Highlighted are molecules displaying novel mechanisms of action that were recently approved for human use or that are now undergoing phase II/III clinical trials. Particular emphasis is placed on the identification of new drugs and drug candidates for the treatment of schizophrenia and major depression. Finally, a specific section is dedicated to neurodegenerative disorders such as Alzheimer’s disease (AD), where pharmacological strategies can significantly differ from the approaches currently adopted in psychotic and affective disorders.

## Schizophrenia

At a mechanistic level, drug treatments for schizophrenia are presently based on the dopamine hypothesis concerning the symptoms of this disorder ^4^. The development of second-generation antipsychotics that began 25 years ago has yielded some advances in terms of efficacy, with some modest improvement in addressing the negative symptoms of this condition, and in tolerability, particularly with regard to extrapyramidal side effects ^5^. However, no antipsychotics display robust effects on the cognitive deficits or impaired social processing that are important components of this disorder ^4^. For years, the limited efficacy of conventional and second-generation agents has led to theories about whether the manipulation of brain targets other than, or in addition to, the dopamine D2 receptor (D2R) may be necessary for treating this disorder and to significantly improve safety and tolerability.

In recent years, the N-methyl-D-aspartate (NMDA) receptor hypothesis of schizophrenia has been validated in preclinical animal models and humans ^6^. According to this theory, the excessive dopamine release in the mesolimbic pathway and the decrease in dopamine release from the mesocortical pathway in the prefrontal cortex, which are responsible for some of the symptoms of schizophrenia, are secondary to a decrease in NMDA receptor control of inhibitory GABAergic neurons. No drugs capable of selectively enhancing NMDA receptor activity in this key brain region have yet been approved for human use. Studies indicate that prodromal and early-in-disease schizophrenic patients have elevated brain glutamate levels compared to healthy controls and ultra-high-risk patients who do not become psychotic ^7^. Alterations in NMDA receptor-mediated excitation of GABAergic neurons indicate that schizophrenia is associated with dysfunctional glutamatergic systems in the prefrontal cortex and limbic regions of the brain. One approach has been to target the glutamatergic system using pomaglumetad methionil, a potent and highly selective orthosteric metabotropic glutamate receptor (mGluR) 2/3 agonist ^8^, but alternative approaches directed at group III mGluRs are also currently studied in preclinical models of schizophrenia ^9^. Preclinical research suggests that by reducing glutamate release, pomaglumetad methionil normalizes heightened glutamate activity in cortical pyramidal neurons ^8^. While pomaglumetad methionil was reported to display beneficial effects on both the positive and the negative symptoms of schizophrenia in initial clinical trials, positive results were not obtained in phase III studies. Analysis of the clinical data suggests that pomaglumetad methionil is most efficacious in early-in-disease schizophrenics (<3 years’ duration) with a known hyperactive glutamatergic pathophysiology ^8^. It is anticipated, therefore, that new antipsychotics acting on the mGluR2/3 will be developed to treat schizophrenic patients in an early phase of the illness with the hope of slowing disease progression and improving prognosis.

The dopamine D3 receptor (D3R) is another pharmacological target that appears to play a prominent role in the pathogenesis of schizophrenia. Unlike the D2R, which has been extensively studied with respect to the symptoms of schizophrenia, little is known about the extent to which changes in D3R and dopamine D4 receptor (D4R) activity contribute to the symptoms of this disorder. It is known that the molecular structure of D3R is very similar to that of D2R and D4R and that in a variety of animal species D2R and D3R share high homology ^10^ and identity in the transmembrane regions, including the binding site ^11^. Such structural similarities make it difficult to design ligands that selectively interact with D3R. This, in turn, has compromised the ability to fully define the localization and functions of this site. A number of approved drugs that were believed to act primarily at the D2R recognition site have now been found to interact with the D3R as well (see 12 for review). Cariprazine is the first example of a D3R partial agonist that occupies this receptor at doses that produce antipsychotic-like effects in preclinical animal models ^13, 14^. Cariprazine displays higher affinity at D3R and a similar affinity at D2 and serotonin 2B (5-HT _2B_) receptors ^13^. In rodents, cariprazine reverses the deficit in novel object recognition that is caused by neonatal administration of phencyclidine ^14^ or by subchronic phencyclidine exposure in adults ^15, 16^. Preclinical and clinical data suggest that by selectively activating D3R, cariprazine may have a positive impact on the cognitive symptoms of schizophrenia. Cariprazine was approved in 2015 by the U.S. Food and Drug Administration for the treatment of schizophrenia ^17^. Further clinical studies with cariprazine and other antipsychotics selectively targeting D3R will be essential to validate the role of D3R as a new pharmacological target for the treatment of cognitive symptoms in schizophrenia.

Another new approach proposed for the treatment of schizophrenia is the development of disease-modifying agents to prevent the full onset of schizophrenia and/or to slow its progression ^4^. Many questions remain as to whether it will be possible to identify drugs that will directly affect the underlying disease process in a way that would delay or prevent disease progression in schizophrenia.

There are also many challenges to consider for designing clinical trials to prove that a given drug candidate can modify the course of this disorder over time. A first step might be to identify young individuals who are at high risk for psychosis. Particular emphasis could be placed on those who are also heavy users of cannabis, as there is an increased risk for schizophrenia in those who abuse highly potent preparations of cannabis (so-called “skunk” variants) containing a high percentage of delta-9-tetrahydrocannabinol (THC) (about 15%) with a scarcity of cannabidiol (CBD 0–1%) ^4, 18^. As a negative allosteric modulator of the cannabinoid 1 (CB1) receptor, CBD ameliorates the psychotogenic effect of THC and may possess antipsychotic properties ^19^. Given these findings, the CB1 receptor has been proposed as a new pharmacological target for the treatment of schizophrenia ^6, 19^. Indeed, there have been reports that 800 to 1,000 mg of cannabidiol per day safely reduces the signs and symptoms of schizophrenia ^19^. However, uncertainty remains about the mechanism of CBD action. Recently, Seeman reported that CBD, like aripiprazole, is a partial agonist at the D2R ^20^. Thus, CBD could be the first molecule of a class of antipsychotics that interact with both the CB1 and the D2R. Ideal candidates for clinical trials with such agents would be high-risk individuals to assess whether CBD dampens the acute psychotic symptoms and cognitive deficits associated with schizophrenia. Three different phase II clinical trials are underway to assess the clinical efficacy of cannabidiol monotherapy in newly diagnosed schizophrenic patients and as adjunctive therapy with conventional second-generation antipsychotics (NCT02088060 and NCT02504151).

## Depression

While currently available antidepressants, such as selective serotonin reuptake inhibitors (SSRIs) and serotonin and noradrenaline reuptake inhibitors (SNRIs), are effective for most patients, approximately 30% of those with major depressive disorder (MDD) fail to respond to these agents ^2^. Cognitive dysfunction represents a distinct biological and clinical dimension in MDD ^21^, with evidence suggesting that the presence of cognitive symptoms in depressed patients can predict a low rate of response to antidepressants and reduced remission rates ^22, 23^. Multimodal drugs, such as vortioxetine, represent a new class of antidepressants. These agents display multiple molecular mechanisms of action in addition to inhibition of the serotonin transporter ^24, 25^.

Vortioxetine is a multimodal antidepressant that is thought to act by inhibition of transmitter reuptake and interactions with various 5-HT receptor sites. This pharmacodynamic profile is well described in the Neuroscience-Based Nomenclature, a new pharmacologically driven classification of psychotropic drugs that reflects current knowledge about the underlying neurobiological characteristics of the disorder, an understanding of the neurotransmitter/molecule/system being modified (“pharmacological domain”), and the mode/mechanism of drug action ^26^. Vortioxetine is not the only example of a multimodal antidepressant, with others, such as vilazodone (a serotonin transport inhibitor and a 5-HT _1A_ receptor partial agonist), having been approved for the treatment of major depression ^27, 28^. Other psychotropic drugs developed in the last 30 years can possess a multimodal pharmacodynamic profile (see e.g. trazodone), but the novelty of this approach is to combine multiple pharmacological actions affecting both monoamine targets and other non-monoaminergic systems (e.g. glutamatergic system) ^24^. The multimodal approach seems to be an interesting approach to target the different biological and clinical dimensions of MDD. Current evidence does not suggest a global greater efficacy of multimodal antidepressants compared to SSRIs or SNRIs but an improved efficacy on specific clinical domains where SSRIs or SNRIs are less effective, as observed with vortioxetine, which displays a specific clinical efficacy in the treatment of cognitive deficits associated with MDD ^25^.

Vortioxetine is a 5-HT _3_, 5-HT _7_, and 5-HT _1D_ receptor antagonist, 5-HT _1B_ receptor partial agonist, 5-HT _1A_ receptor agonist, and an inhibitor of the serotonin transporter ^29^. It is thought to activate the glutamatergic system in rat frontal cortex by blockade of 5-HT _3_ and 5-HT _7_ receptors. It is reported that, as compared to fluoxetine, vortioxetine displays a superior efficacy in aged mice as a treatment for visuospatial memory and depression-like behavior ^30^. The pharmacodynamic profile is consistent with the results of clinical studies indicating that vortioxetine has antidepressant properties as well as positive effects on cognitive function (e.g. memory and executive functioning) ^25^. The most common side effects associated with this drug are nausea, vomiting, and constipation. Vortioxetine is currently studied as a potential therapeutic alternative for patients who fail to respond to SSRIs or SNRIs ^31^.

Drugs, such as ketamine, that are known to affect descending glutamatergic systems represent a new approach for managing treatment-resistant depression (TRD) ^32^. Several well-controlled clinical studies indicate that a single ketamine infusion (0.5 mg/kg) induces a rapid, generally transient, antidepressant effect in addition to small, but significant, increases in psychotomimetic and dissociative symptoms ^33^. The ketamine-induced antidepressant effect occurs within 1 to 2 hours following its intravenous administration and may be sustained for up to 2 weeks ^34^. This rapid onset of action has stimulated studies to explore the possibility that ketamine may represent a life-saving drug for TRD patients at imminent risk of suicide ^35^. Twice- and thrice-weekly administration of ketamine at 0.5 mg/kg maintains antidepressant efficacy for over 2 weeks with no signs of tolerance ^36^.

It is believed that by blocking NMDA receptors on GABAergic interneurons, ketamine causes a rapid, but transient, increase in extracellular glutamate in the prefrontal cortex. At the molecular level, the ketamine-induced blockade of NMDA receptors results in inhibition of elongation factor 2 (eF2) kinase, de-phosphorylation of eF2, and a consequent augmentation of brain-derived neurotrophic factor (BDNF) synthesis ^32^. Preclinical and clinical studies with ketamine have resulted in the identification of new pharmacological targets related to NMDA receptor activation or inhibition, such as the NR2B receptor subunit, and the mammalian target of rapamycin (mTOR), a signaling system that controls synaptic plasticity and appears to be a key element in the antidepressant response to ketamine ^32^. Recent studies also suggest that the pharmacological profile of ketamine is more complex than being a simple NMDA receptor antagonist because this drug also shows a significant affinity for D2R and opioid receptors as well as for monoamine transporters ^37^. Further studies are needed to identify all the molecular mechanisms underlying the rapid-acting antidepressant effects of ketamine. Efforts are now directed towards the development of new antidepressants displaying the efficacy and rapid onset of action of ketamine but lacking its psychotomimetic effects ^34^.

Attempts are also underway to exploit the established clinical value of ketamine and its derivatives, such as S(+) ketamine (esketamine), while reducing their side effect potential by administering them intramuscularly or intranasally at doses (e.g. 0.2 mg/kg) lower than those used for the intravenous studies ^38^. Phase III clinical trials are ongoing to assess the safety and clinical efficacy of intranasal esketamine in patients with TRD (NCT02782104, NCT02133001, and NCT02497287).

An alternative approach to target the glutamatergic system in MDD and mimic ketamine’s effects might be the use of mGluR5-selective antagonists or negative allosteric modulators ^39^. This novel approach stems from the evidence that mGluR5s are functionally involved in the mild modulation of NMDA receptor activity and mGluR5 antagonists exert significant antidepressant effects in animal models of depression by acting as mild NMDA receptor negative modulators ^39^. Therefore, mGluR5s have been recently considered as a new target of novel antidepressants, and basimglurant, a negative allosteric modulator of mGluR5, is now in clinical development for the treatment of MDD (NCT00809562 and NCT01437657).

## Alzheimer’s disease

AD is a neurodegenerative disorder characterized by memory loss, cognitive decline, and neuropsychiatric symptoms that interfere with normal daily activities ^40^. This disorder is associated with the presence of senile plaques containing amyloid β (Aβ), intracellular aggregates of tau protein in neurofibrillary tangles, and progressive neuronal loss. The amyloid cascade hypothesis ^41^ posits that overproduction of Aβ, or failure to clear this peptide, causes AD because of the aggregation of monomeric Aβ species into higher-molecular-weight Aβ oligomers that results in neuronal cell loss ^42^.

Current drug therapies for AD treat only the symptoms, such as memory loss, while having no effect on the progression of the disease. Drugs included in this group are cholinesterase inhibitors (donepezil, rivastigmine, and galantamine), which are approved for treating mild to moderate AD, and memantine, an NMDA receptor antagonist, which is used to treat patients with moderate to severe AD.

Much effort has been expended in developing disease-modifying drugs for the treatment of this condition. Such agents would be able to slow the progression of the pathological changes and their effects would persist even after terminating treatment ^42^. To date, this effort has failed to yield any clinically effective drugs. One of the difficulties in testing such compounds has been the lack of reliable biomarkers identifying patients in the early stage of the disease. For this reason, most clinical trials were conducted in patients in advanced stages of the disease after irreversible damage to the brain had already occurred. However, the criteria for the diagnosis of AD, revised in 2011 by the National Institute on Aging and the Alzheimer’s Association workgroup ^43^, now include biomarkers for identifying AD patients earlier. Such individuals are much better candidates for treatment with disease-modifying drugs ^44^. The development of these biomarkers increases the likelihood of identifying that cohort of patients that is most likely to respond to disease-modifying drugs.

Immunotherapy directed towards Aβ has been considered a promising approach because it would, in theory, decrease the aggregation and brain deposition of Aβ. Tau immunotherapy has also been explored as a possible means for inhibiting disease progression in AD ^45^. Given problems with vaccines, Aβ passive immunotherapy is currently the most popular approach for developing disease-modifying drugs for the treatment of AD ^42, 46^.

As antibody-based immunotherapy against Aβ has so far failed to yield positive clinical results, questions are being raised about the validity of the amyloid hypothesis of AD. However, it remains unknown whether the clinical failures reported for bapineuzumab, gantenerumab, and solanezumab are due to their inability to reduce the formation of Aβ oligomers or because they were tested in inappropriate populations of AD patients ^47, 48^. Unfortunately, encouraging preliminary results obtained with solanezumab have not been confirmed in recent phase III clinical trials ^47, 48^. This drug is, however, still in clinical development in prodromal AD patients (NCT02008357 and NCT02760602).

The recent positive results with aducanumab in prodromal and mild AD in a phase Ib trial ^49, 50^ suggest that the amyloid hypothesis is still consistent with the development of new disease-modifying drugs, based on this hypothesis, in the near future. Aducanumab is the first example of an antibody developed by selecting human B-cell clones triggered by neo-epitopes present in soluble oligomers and insoluble fibrils. That is, only the pathogenic forms of Aβ ^49^ are affected without interfering with Aβ monomers that exert a critical role in maintaining neuronal survival, learning, and memory ^50, 51^. It has been established that aducanumab enters the brain and that 1 year of monthly intravenous infusion reduces brain Aβ in a dose- and time-dependent manner in patients with prodromal or mild AD. The greatest effects are observed at doses of 3 and 10 mg/kg. This effect was accompanied by a slowing of the clinical decline as measured by the Clinical Dementia Rating—Sum of Boxes and Mini Mental State Examination scores. As already observed with solanezumab, amyloid-related imaging abnormalities (ARIA), such as vasogenic edema, indicate a dose-dependent response in AD patients ^49^. The clinical efficacy of aducanumab must be confirmed in the long-term extension phase of this study as well as in the ongoing phase III clinical trials, which may finally validate the amyloid hypothesis in the field of AD.

## Conclusion

As indicated in this report, the pathology-to-drug discovery approach is now being applied for the identification of new drugs for the treatment of psychiatric disorders ( Table 1). This has been responsible, in part, for the identification of new psychiatric medications with novel mechanisms of action (D3R antagonists, vortioxetine, and esketamine). Secondary prevention strategies with glutamatergic agents (mGluR2/3 agonists) or negative allosteric modulators of CB1 (cannabidiol) are under study for the treatment of schizophrenia. If confirmed in ongoing clinical trials, the early results with aducanumab, a possible disease-modifying agent for AD, suggest that the pathology-to-drug discovery approach may be applicable for designing and developing new medications for the treatment of a host of neuropsychiatric disorders.

**Table 1.** Current status of clinical development of new psychotropic drugs for the treatment of neuropsychiatric disorders.

Drug	Indication/field	Putative mechanism	Phase of study of action	Main result and/or status
Pomaglumetad	Schizophrenia	Agonist on mGlu2/3	III	Efficacy only in schizophrenic patients with a duration of illness <3 years
Cariprazine	Schizophrenia	D3/D2/5-HT2B ligand with a preferential affinity for D3R	Approved	Approved
Cannabidiol	Schizophrenia	CB1 negative allosteric modulator D2R partial agonist	II	Ongoing
Vortioxetine	Depression	Multimodal (5-HT receptor agonist/antagonist reuptake inhibitor)	Approved	Approved
Vilazodone	Depression	Multimodal	Approved	Approved
Ketamine (esketamine)	Depression	NMDA antagonist	III	Ongoing
Aducanumab	Alzheimer’s disease	Aβ clearance	III	Ongoing

5-HT, serotonin; Aβ, amyloid beta; CB1, cannabinoid 1; D2R, dopamine 2 receptor; D3R, dopamine 3 receptor; mGlu, metabotropic glutamate; NMDA, N-methyl-D-aspartate

## Abbreviations

5-HT, serotonin; Aβ, amyloid beta; AD, Alzheimer’s disease; CBD, cannabidiol; CB1 receptor, cannabinoid 1 receptor; D2R, dopamine 2 receptor; D3R, dopamine 3 receptor; D4R, dopamine D4 receptor; eF2, elongation factor; MDD, major depressive disorder; mGluR, metabotropic glutamate receptor; mTOR, mammalian target of rapamycin; NMDA, N-methyl-D-aspartate; SNRI, serotonin and noradrenaline reuptake inhibitor; SSRI, selective serotonin reuptake inhibitor; THC, delta-9-tetrahydrocannabinol; TRD, treatment-resistant depression.
